# Distribution of subtypes and immunophenotypic characterization of 1379 cases of paediatric acute leukaemia

**DOI:** 10.12669/pjms.37.3.3552

**Published:** 2021

**Authors:** Saba Jamal, Fatima Meraj, Neelum Mansoor, Sadia Parveen, Ameerah Shaikh, Naeem Jabbar

**Affiliations:** 1 Dr. Saba Jamal (MBBS) Diplomate American Board of Anatomic and Clinical Pathology, Diplomate American Board of Hematology), Haematology Clinical Laboratory, The Indus Hospital, Karachi, Pakistan; 2 Dr. Fatima Meraj (MBBS, MCPS, FCPS), Haematology Clinical Laboratory, The Indus Hospital, Karachi, Pakistan; 3 Dr. Neelum Mansoor (MBBS, FCPS), Haematology Clinical Laboratory, The Indus Hospital, Karachi, Pakistan; 4 Ms. Sadia Parveen (M.Phil.), Indus Hospital Research Center, The Indus Hospital, Karachi, Pakistan; 5 Ameerah Shaikh, Medical Student, Ziauddin Medical College, Karachi, Pakistan; 6 Dr. Naeem Jabbar (MBBS, FCPS). Paediatric Haematology Oncology, The Indus Hospital, Karachi, Pakistan

**Keywords:** Paediatric acute leukemia, Immunophenotyping, Phenotypic aberrant markers, Flowcytometry

## Abstract

**Objectives:**

Acute leukaemia is the most common and highly curable childhood malignancy; subtyping and identification of antigens via immunophenotyping helps in treatment plan as well as minimal residual disease monitoring.

**Methods:**

This retrospective study was conducted at the Haematology section of the clinical laboratories of Ziauddin University Hospital and The Indus Hospital, Karachi conducted at January 1st, 2012 to December 31^st^, 2017. The study included 1379 cases of de novo acute leukemia from 2012 to 2017. Among these, 80% were diagnosed by using four color flowcytometry (FACS Calibur), 9% and 11% via immunohistochemistry on bone marrow trephine biopsy samples and morphological examination respectively.

**Results:**

The mean age of patients was 7.4 ± 4.3 years while male to female ratio was 1.75:1. Lymphoblastic leukaemia accounted for 77.2% and myeloid leukaemia 21.2%. Amongst lymphoblastic lineage, B-ALL was 80.4% while T-ALL was 19.6%. Among the phenotypic expression of B-ALL, CD79a (99.8%) had the highest positivity. In B-ALL, CD13 (29.8%) was the most common aberrant myeloid marker. Aberrant expression of CD79a observed in 11.1% of T-ALL cases. In non APL AML, aberrant expression of CD79a and CD19 was observed in 6.6% and 5.5% of cases respectively.

**Conclusion:**

Overall immunophenotypic profile, expression of aberrant phenotypes and subtype distribution in our patients was similar to international literature except for a relatively high frequency of T-ALL which was discordant from the western data.

## INTRODUCTION

Acute leukaemia is a heterogeneous group of malignancies with varying clinical, morphologic, immunologic, and molecular characteristics.^1^,^2^ Distinction between lymphoid and myeloid leukaemia, made by immunophenotyping, is crucially important to identify subtypes as they carry predictable prognosis and warrant specific therapy. Immunophenotypic characterization of acute leukaemia by flowcytometry or immunohistochemistry has resulted in precise delineation.^3^,^4^ Detailed understanding of these phenotypic patterns of differentiation allows for more precise classification of acute leukemia than does morphology alone.^5^

Moreover, identification of aberrant antigen expression is important in characterizing neoplastic population among non-neoplastic counterparts. Aberrant phenotypes aid in subsequent testing of minimal residual disease during the course of treatment.

A recently published study demonstrates incidence, prognostic factors and outcomes of childhood acute myeloid leukemia (AML).^6^ Unfortunately, no large study is published from Pakistan which demonstrates the distribution of childhood acute leukaemia. It is important to know the trends in our population and identify differences from other regional and western populations with respect to acute leukaemia subtypes and immunophenotypic profile. This study, the largest in the country, is aimed to report the subtypes of childhood acute leukaemia, antigen profile and aberrant antigen expression according to our demographics. Sequential data analysis was carried out on all consecutive samples received from paediatric hematology oncology unit of The Indus Hospital, Karachi Pakistan, in order to identify immunophenotypic patterns in our paediatric population. No patient was excluded from the study resulting in a very large sample size, which enhanced the accuracy and precision of the results.

## METHODS

**Fig.1:**
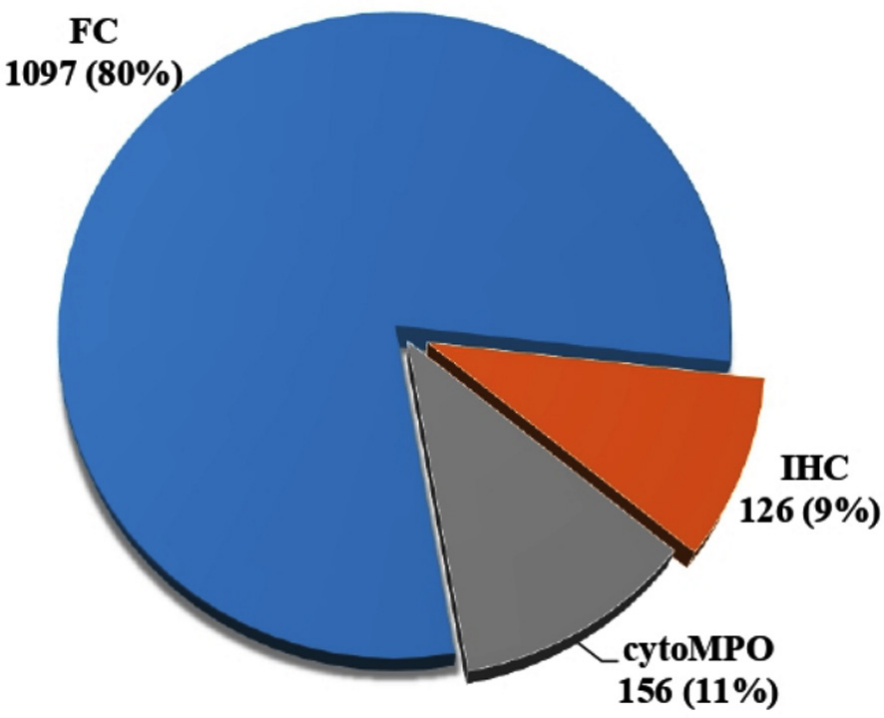
Distribution of modalities used for diagnostic purpose. FC: Flowcytometry, IHC: Immunohistochemistry, cytoMPO: Cytochemical Myeloperoxidase

This retrospective study was conducted at the Haematology section of the clinical laboratories of Ziauddin University Hospital and The Indus Hospital, Karachi. The study was reviewed by the ethical board of Indus Hospital (IRB No: IRB_IRD_2018_10_005). The study included 1379 newly diagnosed consecutive cases of suspected de novo acute leukaemia (≥20% blasts in blood or bone marrow; lineage to blast population is assigned as per WHO 2008/2016/2017 criteria) from January 1st, 2012 to December 31^st^, 2017. Inclusion criteria were patients belonging to either gender and below 18 years of age. Peripheral blood and/or bone marrow aspirate samples in EDTA tube along with trephine biopsy were collected from the paediatric haematology-oncology unit of The Indus Hospital and transported to the haematology department of Ziauddin University Hospital, clinical laboratories for analysis. Samples were tested on haematology analyzer Coulter LH500 for white blood cells (WBC), Leishman stained slides were prepared in parallel for morphologic evaluation of blast percentage. Cytochemical myeloperoxidase (cytoMPO) staining was also performed on all peripheral blood samples and/or bone marrow aspirates; samples with ≥3% positive blasts were considered positive for cytoMPO and classified based on morphological classification of AML as per WHO classification. Immunophenotyping by 4-colour flowcytometry using FACS Calibur flowcytometer (Becton Dickinson, Biosciences) and/or immunohistochemistry on trephine biopsies was performed on all cytoMPO negative samples as well as on samples with less than 3% cyto MPO positivity (Fig.1). The antibodies used for flowcytometry (BD Biosciences) were as follows:

CD45, CD34, TdT, cytoMPO, cyCD3, cyCD79a, CD19, CD22, CD20,CD10, CD4, CD8, CD5, CD7, CD13, CD33,CD11c, HLA-DR, CD15, CD36,CD64, CD117, cyCD61, Glycophorin A, CD58, CD66 and CD9.

Flowcytometric analysis was carried out using Paint-A-Gate software. Samples with low WBC, hemodiluted/dry tap bone marrow aspirates were immunophenotyped by immunohistochemistry (IHC) performed on bone marrow trephine biopsies. The panel of IHC included TdT, CD34, CD117, CD79a, CD20, CD3, cytoMPO, CD64, CD163, Glycophrin A, CD61, CD4, CD8, CD5, CD7 and CD10.

Statistical analysis was performed using SPSS version 24.0. Numbers with percentages were used for discrete variables and mean ± standard deviation was used for normally distributed continuous variables. Chi-square test was used to assess an association between disease entity and pre-defined age groups. Significance was assessed at P ≤ 0.05.

## RESULTS

### Patient’s characteristics:

A total of 1379 paediatric acute leukaemia cases were included in the study. Distribution of modalities used for diagnosis is shown in Fig.1. Male to female ratio was 1.75:1 with male preponderance in all acute leukaemia cases. In B-ALL, T-ALL and AML male to female ratio was 1.5:1, 3.8:1 and 1.5:1 respectively. Age distribution of B-ALL and T-ALL showed that 75.7% and 62.7% were between 1 and 10 years respectively, while AML occurred in 63.4% in this age group (Table-I). Statistically significant difference was observed between ALL and AML (p=0.003) as well as in B-ALL and T-ALL (p=0.00) in the 3 age groups.

**Table I:** Clinical Characteristics of acute leukaemia.

Clinical characteristics	Total patients n=1379	<1 year n=25 (1.8%)	1-10 years n=975 (70.7%)	>10 years n=379(27.5%)
***Age (years)***				
Mean Age at the time of Diagnosis ± SD	7.4±4.3	0.6±0.3	5.4±2.7	13.2±1.6
Median (Range)	7(0.25-18)	0.7(0.25-0.9)	5(1-10)	13(10.3-18)
***Gender, n (%)***				
Male	878(63.7)	17(68)	614(63)	247(65.2)
Female	501(36.3)	8(32)	361(37)	132(34.8)
***Disease entity, n (%)***				
Lymphoblastic Leukaemia	1064(77.2)	15(1.4)	776(73.0)	273(25.6)
B-ALL	855/1064(80.4)	13(1.5)	647(75.7)	195(22.8)
T-ALL	209/1064(19.6)	0(0)	131(62.7)	78(37.3)
Myeloid Leukaemia	292(21.2)	10(3.4)	185(63.4)	97(33.2)
Non APL	278/292(95.2)	10(3.6)	178(64)	90(32.4)
APL	14/292(4.8)	0	7(50)	7(50)
MPAL	13(0.9)	0	7(54)	6(46)
AL (not sub classified)	10(0.7)	0	7(70)	3(30)

ALL: Acute lymphoblastic leukemia, AL: Acute Leukaemia MPAL: Mixed phenotypic acute leukaemia.

### Lymphoblastic leukemia:

Among the B-ALL, CD79a and CD19 were expressed in 99.8% and 99% cases, respectively whereas expression of CD20 was observed in 59.4% cases. There were 5.3% of cases negative for CD10 (Table-II) and 2.8% were negative for both TdT and CD34 (Table-III).

**Table II:** Antigen expression in B-ALL (n=855), T-ALL (n=209) and Non APL AML (n=261, excluding AML-not otherwise classified).

Antigens	Expression, n/N (%)		

	B-ALL	T-ALL	Non APL AML
CD 45	669/771(86.8)	185/186(99)	78/79(98.7)
TdT	802/843(95)	171/206(83)	24/98(24.5)
CD34	519/833(62.3)	51/200(25.5)	68/123(55.3)
CD117	3/150(2)	3/91(3.3)	74/109(67.8)
CD79a	814/815(99.8)	20/180(11.1)	6/90(6.6)
CD 19	769/776(99)	0/187	5/90(5.5)
CD22	590/642(92)	2/166(1.2)	3/73(4.1)
CD20	434/731(59.4)	0/161	0/65
CD10	792/836(94.7)	95/177(53.6)	2/63(3.2)
CD3	0/829	209/209(100)	0/114
CD5	-	166/184(90.2)	23/50(46)
CD4	3/163(1.8)	162/204(79.4)	23/50(46)
CD8	-	109/203(53.7)	0/24
CD7	-	22/24(91.7)	-
CD4/8 co-expression	-	102/203(50.2)	-
CD4/8 double negative	-	35/203(17.2)	-
CD4^pos^/CD8^neg^	-	59/172(34.3)	-
CD8^pos^/CD4^neg^	-	7/203(3.4)	-
MPO (Cyto/IHC/FC)	0/685	0/196	202/261(77.4)
CD13	194/651(29.8)	13/161(8.1)	91/106(85.8)
CD33	122/708(17.2)	13/173(7.5)	97/106(91.5)
CD15	101/496(20.3)	9/109(8.3)	44/70(62.8)
HLADR	179/183(97.8)	15/61(24.6)	41/62(66.1)
CD64	-	-	33/90(36.7)
CD36	-	-	10/54(18.5)
CD11C	-	-	15/41(36.5)
cyCD61	-	-	9/88(10.2)
GlyA	-	-	2/72(2.7)
CD66	191/619(30.8)	0/142	10/56(17.8)
CD58	332/343(96.8)	80/99(80.8)	33/38(86.8)
CD9	393/410(95.8)	33/100(33)	24/43(55.8)

CD: Cluster of differentiation, Cyto: Cytochemistry, FC: Flowcytometry, HLA DR: Human leukocyte antigen, IHC: Immunohistochemisty, MPO: Myeloperoxidase, n/N: No. of antigen postive/ Total no. of cases tested, TdT: Terminal deoxynucleotidyltransferase, GlyA: Glycophorin A

**Table III:** Incidence of TdT-negative, CD34-negative and TdT/CD34 Double-negative ALL in present study and reported in other studies.

	Antigens	Present study results, n/N (%)	Other studies
	TdT^-^	41/843 (4.8)	11.4%^25^, 38%^22^
B-ALL	CD34^-^	314/833 (37.6)	41%^26^, 44%^22^
	CD34^-^/TdT^-^	23/826 (2.8)	5.7%^25^
	TdT^-^	35/206 (16.9)	28.5%^25^, 57%^22^
T-ALL	CD34^-^	149/200 (74.5)	78%^22^
	CD34^-^/TdT^-^	22/198 (11)	0.5%^27^

n/N: No. of cases lacking the tested expression/ Total no. of cases tested

Markers used to characterize T-ALL (Table-II) included intracytoplasmic CD3 (100%) with highest expression rate followed by CD5 (90.2%). Co-expression of both CD4 and CD8 (CD4+/CD8+) was observed in 50.2% cases while 17.2% of T-ALL cases were CD4 and CD8 double negative (CD4-/CD8-). In T-ALL, 11% (22/198) cases were negative for both TdT and CD34 (Table-III). Expression of CD45 was observed in 86.8% and 99% of B-ALL and T-ALL cases respectively.

In B-ALL, CD13 (29.8%) was the most common aberrant myeloid marker followed by CD15(20.3%) and CD 33 (17.2%), while CD15, CD 13 and CD 33 were expressed in 8.3%, 8.1% and 7.5% of T-ALL cases respectively. In T-ALL, 11.1% of cases aberrantly expressed CD79a and 3.3% cases showed CD117 positivity.

### Acute myeloid leukaemia:

**Fig.2:**
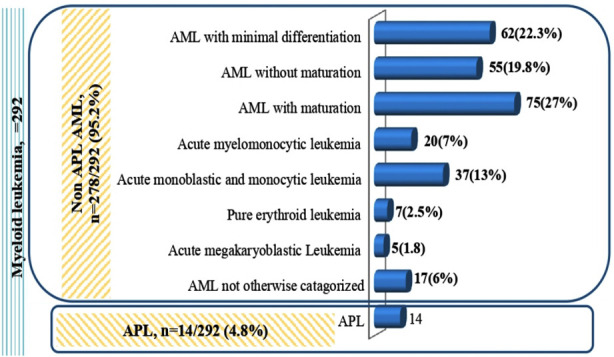
Distribution of AML, not otherwise specified (NOS) and APL cases according to WHO classification 2008/2017.

There were 278 non APL-AML cases, among which, 261 were classified according to WHO classification while 17 cases could not be further sub classified. Acute pro-myelocytic leukaemia (APL) accounted for 4.8% of AML cases. Mean (±SD) age of AML patients was 7.8 (±4.5) years. Most common subtype in non-APL AML group (Fig.2) was AML with maturation (n=75/278, 27%). In Non APL AML cases, CD79a and CD19 were expressed in 6.6% and 5.5% of cases respectively (Table-II).

### Mixed phenotypic leukaemia (MPAL):

Frequency of MPAL was found to be 0.9% (n=13), which included B/myeloid (n=6), T/myeloid (n=4), and B/T lymphoid (n=3). Mean (±SD) age was 10.5(±3.4) years in MPAL group. Expression of TdT and CD34 was observed in 92.3% and 38.4% cases respectively. All MPAL cases were CD45 positive (n=13, 100%). In B/Myeloid cases, CD19 (6/6, 100%), CD79a (6/6, 100%), CD20 (1/7, 14.2%), CD10 (5/6, 83.3%), CD13 (1/6, 16.6%), CD33 (2/6, 33.3%) and cytoMPO (6/6, 100%) expression was observed. In T/Myeloid, CD3 (4/4, 100%), cytoMPO (4/4, 100%), CD13 (3/4, 75%) and CD15 (1/3, 33.3%) were positive. In mixed B and T lymphoid, all cases showed CD19 (3/3, 100%), CD79a (2/2, 100%), CD20 (2/3, 66.6%), CD3 (3/3, 100%) and CD10 (3/3, 100%) expression. In mixed B/T lymphoid aberrant expression of myeloid markers i.e CD13 (2/3, 66.6%) and CD33 (1/2, 50%) were also observed.

## DISCUSSION

Despite the fact that childhood acute leukaemia is one of the most common^7^,^8^ and curable^9^ malignancies, data published from Pakistan is limited describing subtype distribution and detailed immunophenotypic characterization. To the best of our knowledge, the present data from Pakistan is one of the largest studies (1379 cases in six years) in which all consecutive cases of acute leukaemia presenting to the paediatric haematology-oncology department were categorized by extensive immunophenotyping. Despite high incidence of leukemia, currently no registry exist in our country to demonstrate actual data. This study represents the true demographics and distribution of the subtypes of acute leukaemia in the paediatric population of Pakistan. Most of the cases were diagnosed by advanced flowcytometry technique utilizing extensive panel of markers. These panels not only comprised of lineage specific markers as well as other large number of markers that can be utilized in subsequent minimal residual disease (MRD) testing. This immunophenotypic profile will definitely aid to design panels for MRD, which play an important role in risk stratification of these patients.

In the present study, patients were categorized into three age groups and results showed that highest number of acute leukaemia cases was found in age group 1-10 years with male gender predominance observed in all subtypes. Published literature from Pakistan as well as other regional studies stated similar findings.^1^,^10^

A study conducted by Amna et al from Pakistan reported 83.3% ALL^11^, two studies from India reported 76.9 and 77.84%^12^,^13^ while the present study has reported 77.2% cases of ALL. Similar Pakistani study reported 14.7% AML cases^11^ however the incidence of AML was slightly higher in Indian studies^12^,^13^ and was similar to present study. In terms of MPAL Indian studies reported 1.1-2.3% while our study had slightly low incidence i.e. 0.9%. However, reports from the western countries showed contradictory results from as low as 0.3% to as high as 4.2%.^14^

In our study, low incidence of MPAL may be due to institutional policy i.e. all cytoMPO negative acute leukaemia cases were analyzed by flowcytometry, while only selective cytoMPO positive cases were tested by flowcytometry.

Our study reported incidence (80.4%) of B-ALL which was similar to other Pakistani studies (78.5-87%)^10^,^15^ as well as an Indian study by Madhumathi et al.^16^ However studies from West^10^,^14^,^17^ reported between 72.9 and 91% frequency. Among lymphoblastic leukaemia, 13-23% of T-ALL cases were reported in other studies from Pakistan in last 10 years^10^,^11^,^15^,^18^,^19^ while similar distribution was reported from India^12^,^13^ with concordant distribution reported in the present study. Studies from West reported a relatively lower frequency. Lack of expression of immature markers in this study and comparison to other studies is shown in Table-III.

B-ALL cases showed expression of CD79a (99.8%) and CD19 (99%), which is consistent with published literature^17^ and WHO also assigned CD19 with co-expression of CD79a, CD22 and CD10 for B-lineage.^2^ Studies reported CD19 as the most sensitive marker in diagnosing B-ALL,^20^,^21^ whereas Tong et al. reported CD79a as the most often expressed antigen^17^ in concordance to our study. This however lacks specificity as aberrant expression of B-cell antigen CD79a was observed in 11.1% cases of T-ALL in this study, which is consistent to the reported literature.^22^

Negativity for CD20, a B-cell specific marker was observed in 61.8% of B-ALL cases reported by Tong et al.^17^ which is slightly lower in the current study (297/731, 40.6%) while another study by Seegmiller et al. reported 33.8% CD20 negative cases.^23^

Negativity for CD45 also called leukocyte common antigen was observed in 13.2% B-ALL cohort and was comparable to Seegmiller et al.^23^ Absence of CD10 expression was observed in 5-18.8% reported in eastern and western studies^17^,^23^ and similar frequency (5.3%) was reported in current study.

In our study all T-ALL cases were cyCD3 positive. It is well known fact that cyCD3 is the best marker for T-ALL cases. In our study, CD4/CD8 co-expression was seen in 50.2% cases, CD4/CD8 double negativity in 17.2%, CD4^pos^/CD8^neg^ in 34.3% and CD8^pos^/CD4^neg^ in 3.4% T-ALL cases. Gupta at al. reported CD4/CD8 co-expression in 39.3% cases, CD4/CD8 double negativity in 32.8%, CD4^pos^/CD8^neg^ in 21.3% and CD8^pos^/CD4^neg^ in 6.6% cases.^13^

A wide range of aberrant expression of myeloid antigens including CD13 and CD33 in B-ALL and T-ALL cases reported in both eastern and western literature.^13^,^17^,^22^,^23^ While in present study CD13 (29.8%) was the most common aberrant myeloid marker followed by CD15 (20.3%), and CD 33 (17.2%) in B-ALL. T-cell antigen (CD4) aberrant expression was reported by Seegmiller et al. in 2.2% B-ALL children^23^, which was 1.8% in the present study.

Among the subtypes of AML, the most frequent was AML with maturation similar to the reported literature.^19^ Most published data indicated predominance of acute myeloid leukaemia with and without maturation similar to our study. TdT is a precursor lymphocyte marker, aberrantly expressed in AML. In this study, TdT was expressed in 24.5% of AML cases. B-cell antigens CD79a and CD19 were also expressed in 6.6% and 5.5% cases of AML. This was less frequent than published literature.^24^

### Limitations of the study:

Cytogenetics is the major limitation of this study, as we do not have established karyotyping and mutation analysis which is an important variable according to current WHO Classification. Moreover, due to constraint resources, we could not perform flowcytometric testing on some MPO positive cases that is another deficiency in this study.

## CONCLUSION

On the basis of this large cohort, we can conclude that distribution of acute leukaemia subtypes in our population is close to published literature from Pakistan and India. Aberrant expression of antigens is a well-documented phenomenon and have no therapeutic implications however utilized in minimal residual disease analysis. Prognostic significance of these aberrancies should be evaluated through further studies by correlating with cytogenetics and clinical outcome.

### Authors’ Contributions:

**SJ:** conceived, designed and critically reviewed the manuscript. She is also the principal investigator and will be responsible for accuracy of the work/data.

**FM & NM:** did interpretation of all cases.

**AS, SP & NJ:** did data collection.

**NM, SJ & SP:** did writing of manuscript

**NJ & AS:** did editing and statistical analysis.

**FM & NJ:** did critical review and final editing.

**SJ, FM & NM:** did review and final approval of manuscript.

All authors read and approved the final manuscript.

## References

[1] Bachir F, Bennani S, Lahjouji A, Cherkaoui S, Harif M, Khattab M (2009). Characterization of acute lymphoblastic leukemia subtypes in moroccan children. Int J Pediatr.

[2] Swerdlow S, Campo E, Harris NL, Jaffe E, Pileri S, Stein H (2017). WHO classification of tumours of haematopoietic and lymphoid tissues (revised 4th edition). IARC:Lyon.

[3] Jaffe ES (2001). Pathology and genetics of tumours of haematopoietic and lymphoid tissues:Iarc.

[4] Stam RW, den Boer ML, Pieters R (2006). Towards targeted therapy for infant acute lymphoblastic leukaemia. Br J Haematol.

[5] Dworzak MN, Buldini B, Gaipa G, Ratei R, Hrusak O, Luria D (2018). AIEOP-BFM consensus guidelines 2016 for flow cytometric immunophenotyping of pediatric acute lymphoblastic leukemia. Cytometry B Clin Cytom.

[6] Ghafoor T, Khalil S, Farah T, Ahmed S, Sharif IJCR (2020). Prognostic Factors in Childhood Acute Myeloid Leukemia;Experience from A Developing Country. Cancer Rep.

[7] Nasim N, Malik K, Nauman A Malik, Mobeen S, Awan S, Mazhar N (2013). Investigation on the prevalence of Leukaemia at a tertiary care hospital, Lahore. Biomedica.

[8] Bajwa M, Tahir A, Manzoor I, Khan S, Bakkar M, Mubashir M (2017). Epidemiological distribution of pediatric oncology in Lahore, Pakistan. Biomedica.

[9] Pui C-H, Yang JJ, Bhakta N, Rodriguez-Galindo C (2018). Global efforts toward the cure of childhood acute lymphoblastic leukaemia. Lancet Child Adolesc Health.

[10] Fadoo Z, Nisar I, Yousuf F, Lakhani LS, Ashraf S, Imam U (2015). Clinical features and induction outcome of childhood acute lymphoblastic leukemia in a lower/middle income population:A multi-institutional report from Pakistan. Pediatric Blood Cancer.

[11] Jawaid A, Arif K, Amjad N (2017). Clinical Presentations of Acute Leukemia in Pediatric Emergency Department of Pakistan. Bone.

[12] Gujral S, Badrinath Y, Kumar A, Subramanian P, Raje G, Jain H (2009). Immunophenotypic profile of acute leukemia:critical analysis and insights gained at a tertiary care center in India. Cytometry Part B:Clin Cytom.

[13] Gupta N, Pawar R, Banerjee S, Brahma S, Rath A, Shewale S (2019). Spectrum and immunophenotypic profile of acute leukemia:a tertiary center flow cytometry experience. Mediterr J Hematol Infect Dis.

[14] Noronha EP, Marinho HT, Thomaz EBAF, Silva CA, Veras GLR, Oliveira RAG (2011). Immunophenotypic characterization of acute leukemia at a public oncology reference center in Maranhão, northeastern Brazil. Sao Paulo Med J.

[15] Iqbal Z (2014). Molecular Genetic Studies on 167 Pediatric ALL Patients from Different Areas of Pakistan Confirm a Low Frequency of the Favorable Prognosis Fusion Oncogene TEL-AML1. Asian Pacific J Cancer Prevent.

[16] Madhumathi DS, Prasannakumari Jayadeva Naik, Appaji L, Aruna kumari B, V LD (2015). Optimised panel for characterisation of pediatric ALL in a tertiary cancer centre from a developing country:Initial experience with literature review. Int J Human Pathol Res.

[17] Tong H, Wang Q, Lu C, Liu Z, Hu Y (2011). Immunophenotypic, cytogenetic, and clinical features of 207 cases of childhood acute lymphoblastic leukemia in China. J Pediatr Hematol/Oncol.

[18] Tipu HN, Muhammad MB, Altaf C, Noman M, Malik HS (2018). Spectrum of acute leukemias and aberrant markers expression based on flowcytometry in a tertiary care centre. Pak Armed Forces Med J.

[19] Khan S, Mir A, Khattak B, Rehman A, Zeb A (2017). Childhood Leukemias in Khyber Pakhtunkhwa and Afghan Children Visiting to Hayatabad Medical Complex Hospital. Arch Can Res.

[20] Belurkar S, Mantravadi H, Manohar C, Kurien A (2013). Correlation of morphologic and cytochemical diagnosis with flowcytometric analysis in acute leukemia. J Cancer Res Ther.

[21] Bhattacharyya D, Das S, Sethy S, Singh SC, Mohanty R Study of Clinico-hematological and Immunophenotypic Profile in Adult Patients with Acute Lymphoblastic Leukemia in Eastern India. J Sci Res Rep.

[22] Supriyadi E, Veerman AJ, Purwanto I, Cloos J (2012). Myeloid antigen expression in childhood acute lymphoblastic leukemia and its relevance for clinical outcome in indonesian ALL-2006 protocol. J Oncol.

[23] Seegmiller AC, Kroft SH, Karandikar NJ, McKenna RW (2009). Characterization of immunophenotypic aberrancies in 200 cases of B acute lymphoblastic leukemia. Am J Clin Pathol.

[24] Hamid GA, MA A (2019). Aberrant Antigen Expression in Patients with Acute Leukemia. EC Clin Med Case Rep.

[25] Yasmeen S, Rajkumar A, Grossman H, Szallasi A (2017). Terminal deoxynucleotidyl transferase (TdT)-negative lymphoblastic leukemia in pediatric patients:incidence and clinical significance. Pediatr Develop Pathol.

[26] Supriyadi E, Veerman AJ, Sutaryo S, van de Ven P, Cloos J (2012). Detection of CD10, CD34 and their combined expression on Childhood Acute Lymphoblastic Leukemia and the association with clinical outcome in Indonesia. J Cancer Ther Res.

[27] Wang Y-Z, Hao L, Chang Y, Jiang Q, Jiang H, Zhang L-P (2018). A seven-color panel including CD34 and TdT could be applied in>97% patients with T cell lymphoblastic leukemia for minimal residual disease detection independent of the initial phenotype. Leuk Res.

